# Brain Connectivity Patterns Dissociate Action of Specific Acupressure Treatments in Fatigued Breast Cancer Survivors

**DOI:** 10.3389/fneur.2017.00298

**Published:** 2017-06-23

**Authors:** Richard E. Harris, Eric Ichesco, Chelsea Cummiford, Johnson P. Hampson, Thomas L. Chenevert, Neil Basu, Suzanna M. Zick

**Affiliations:** ^1^Department of Anesthesiology, University of Michigan, Ann Arbor, MI, United States; ^2^Neuroscience Graduate Program, University of Michigan, Ann Arbor, MI, United States; ^3^Department of Radiology, University of Michigan, Ann Arbor, MI, United States; ^4^Department of Epidemiology, University of Aberdeen, Aberdeen, United Kingdom; ^5^Department of Family Medicine, University of Michigan, Ann Arbor, MI, United States; ^6^Nutritional Sciences, University of Michigan, Ann Arbor, MI, United States

**Keywords:** cancer survivors, acupressure, fatigue, connectivity, spectroscopy

## Abstract

Persistent fatigue is a pernicious symptom in many cancer survivors. Existing treatments are limited or ineffective and often lack any underlying biologic rationale. Acupressure is emerging as a promising new intervention for persistent cancer-related fatigue; however, the underlying mechanisms of action are unknown. Our previous investigations suggested that fatigued breast cancer survivors have alterations in brain neurochemistry within the posterior insula and disturbed functional connectivity to the default mode network (DMN), as compared to non-fatigued breast cancer survivors. Here, we investigated if insula and DMN connectivity were modulated by self-administered acupressure by randomizing breast cancer survivors (*n* = 19) to two distinct treatments: relaxing acupressure or stimulating acupressure. All participants underwent proton magnetic resonance spectroscopy of the posterior insula and functional connectivity magnetic resonance imaging at baseline and immediately following 6 weeks of acupressure self-treatment. As compared to baseline measures, relaxing acupressure decreased posterior insula to dorsolateral prefrontal cortex connectivity, whereas stimulating acupressure enhanced this connectivity (*p* < 0.05 corrected). For relaxing but not stimulating acupressure, reduced connectivity was associated with sleep improvement. In addition, connectivity of the DMN to the superior colliculus was increased with relaxing acupressure and decreased with stimulating acupressure, whereas DMN connectivity to the bilateral pulvinar was increased with stimulating and decreased with relaxing acupressure (*p* < 0.05 corrected). These data suggest that self-administered acupressure at different acupoints has specificity in relation to their mechanisms of action in fatigued breast cancer survivors.

## Introduction

There are over three million breast cancer survivors in the United States (1). Breast cancer survivors often report symptoms including fatigue, sleep disturbance, pain, depression, and cognitive impairment (2). These symptoms can be persistent, disabling, and costly to individuals and society. Of these, fatigue is considered to be the most bothersome and also potentially the most difficult to treat (3). Numerous theories of fatigue have been proposed in cancer survivors (4), however, given the co-occurrence of other symptoms (pain, sleep disturbance, mood, and cognitive impairment) the possibility that central neurobiological factors may play a key pathologic role has recently gained traction (5).

Previously, our group found that persistently fatigued breast cancer survivors, as compared to age-matched, non-fatigued breast cancer controls, displayed increases in the concentration of certain brain metabolites within the posterior insula. Specifically, insular creatine to total creatine (Cr/tCr) levels and insular concentrations of glutamate + glutamine (Glx), as assessed by proton magnetic resonance spectroscopy (^1^H-MRS), were significantly higher in survivors with persistent fatigue (6). We reasoned that since the posterior insula is a multimodal sensory processing region and it is involved in interoception (7, 8) (awareness of internal bodily processes), dysfunction in this structure may have led to an altered perception of a person’s sense of fatigue. Moreover, increased levels of brain metabolites in the posterior insula in fatigued survivors may reflect altered energy reserves (Cr is a molecule involved in ATP generation) and/or heightened excitability (glutamate is an excitatory neurotransmitter), both of which might also impact insular function and consequently sensory perception leading to fatigue. Consistent with this notion, we also found different brain functional connectivity patterns between breast cancer survivors with and without persistent fatigue as assessed by functional connectivity magnetic resonance imaging (fcMRI) (9). Specifically, individuals with fatigue showed greater resting connectivity between the brain’s default mode network [DMN; a constellation of brain areas involved in self-referential thinking (10, 11)] and the superior frontal gyrus (an area of the brain involved in self-awareness), and less connectivity between the DMN and brainstem structures including the superior colliculus (SC)/periaqueductal gray (PAG). Based on these results, we hypothesized that altered neurochemistry within the insula and aberrant brain connectivity to the DMN may play a role in the pathology of fatigue and potentially other symptoms in these individuals.

The lack of understanding of the precise mechanistic factors that lead to the development of persistent fatigue and other symptoms in cancer survivors may contribute to the relative dearth of effective treatments for these individuals. One emerging self-help treatment that shows promise in treating cancer-related fatigue is acupressure. Acupressure is a component of traditional East Asian medical practice that involves the stimulation of specific points (acupoints) on the body to regulate function (12). Prior studies have suggested that acupressure may be effective at relieving fatigue and other symptoms in cancer survivors (13, 14). Recently, we performed the largest trial to date of self-administered acupressure (15). In this study, breast cancer survivors with persistent fatigue were randomized to either relaxing acupressure (designed to improve sleep quality), stimulating acupressure (designed to improve daytime activity), or usual care (whatever treatments their physicians recommended for their fatigue). These two acupressure treatments were hypothesized to act differently on ameliorating fatigue: relaxing acupressure was thought to enhance sleep thereby improving subsequent fatigue levels, whereas stimulating acupressure was designed to improve daytime alertness (less fatigue). Interestingly, only relaxing acupressure improved sleep and function. Their differential effects suggest that these two formulas may be unique.

In alignment with current research priorities (16), the goal of this investigation was to determine if these distinct self-administered acupressure formulas (relaxing and stimulating acupressure) have differing effects on the brain in breast cancer survivors. As the concentrations of posterior insular Glx and Cr/tCr are higher in fatigued as compared to non-fatigued breast cancer survivors, we hypothesized that our two acupressure formulas may actually impact these posterior insula neurometabolites differently (one may target Glx and the other Cr/tCr). Furthermore, we did not explore insular connectivity directly *per se* in our previous studies, and given the central role of posterior insula neurometabolites, we reasoned that acupressure treatment may also impact insular connectivity. Finally, since altered DMN connectivity was also observed in this population (9), we hypothesized that stimulating and relaxing acupressure may have differing influences on DMN connectivity. If true, these data could support the theory that these two acupressure formulas have a differential influence on brain neurochemistry and functional connectivity suggesting potentially unique mechanisms of action.

## Materials and Methods

### Participants

19 female breast cancer survivors who had participated in a previously reported clinical trial of acupressure versus usual care for persistent cancer-related fatigue were studied [(15), ClinicalTrials.gov identifier NCT01281904]. The protocol complied with the Code of Ethics of the World Medical Association (Declaration of Helsinki) and was approved by the University of Michigan Medical School, Michigan State University, and Michigan Department of Public Health Institutional Review Boards. All participants provided written informed consent. Details of eligibility and inclusion criteria have been previously reported (17). In brief, eligible women had to report persistent fatigue starting on or after their cancer diagnosis and score ≥ 4 on the Brief Fatigue Inventory (BFI) (18). They also had to be cancer-free and have completed cancer treatments, except hormone therapy, ≥12 months prior. Women were ineligible if they had untreated major depressive disorder, other fatigue-causing comorbidities, cancer diagnosis other than breast cancer or skin cancer within the previous 10 years, were planning on changing or starting a new medication during the study, were taking any medications for insomnia, had contraindications for magnetic resonance imaging (e.g., metal implants), or had received acupuncture or acupressure within the previous 6 months.

### Trial Design

All participants enrolled in a double-blind randomized trial comparing self-administered relaxing acupressure to stimulating acupressure once daily for 6 weeks. The study involved five research visits: screening, baseline, 3-week, 6-week (end of treatment), and 10-week (end of wash-out phase; not analyzed here). ^1^H-MRS and fcMRI were performed once at baseline prior to randomization and then at the conclusion of treatment (week 6).

### Interventions

At baseline, women were taught to self-administer acupressure by a trained acupressure educator (17). The acupressure educators were taught by one of the study’s principal investigators (Richard E. Harris), a National Certification Commission for Acupuncture and Oriental Medicine trained acupuncturist. This training included a 30-min session where educators were taught point location, stimulation techniques, and pressure intensity.

Relaxing acupressure was made up of five points: *Yin tang, Anmian*, heart 7, spleen 6 (SP-6), and liver 3. Four acupoints were performed bilaterally with *Yin tang* done centrally. Stimulating acupressure points consisted of *Du 20*, conception vessel 6 (CV-6), large intestine 4, stomach 36, SP-6, and Kidney 3. Points were administered bilaterally except for *Du 20*, and CV-6, which were done centrally. Acupoint locations are shown in our previously published article that also provides more detail about our acupressure interventions (15, 17). Participants were told to perform acupressure once per day and to stimulate each point in a circular motion for 3 min. A description of the assessments for fidelity of both acupressure educators and participants has been previously reported (19).

### Clinical Outcome Measures

Clinical outcomes were assessed at baseline prior to treatment and then at week 6 following acupressure. Fatigue was assessed with the BFI (18), a scale validated in cancer patients with an alpha coefficient exceeding 0.95, which correlates well with other fatigue measures (20). The BFI assesses severity and impact of fatigue in cancer patients over the past 24 h. The instrument consists of nine items, each measuring fatigue on a 0–10 scale, and is calculated from the mean of completed items. Scores of ≥4 indicate clinically relevant fatigue (18). To assess sleep quality, the 19-item Pittsburgh Sleep Quality Index (PSQI) was used. It evaluates sleep disturbance over the past month. PSQI yields a global score that has a Cronbach’s alpha of 0.81 (21). In women with breast cancer, a score of ≥8 suggests poor sleep quality (21). Women in the acupressure arms were also given a study logbook in which to record adherence to acupressure treatments (data not analyzed).

### Neurobiological Outcomes

#### Proton Magnetic Resonance Spectroscopy

Neuroimaging outcomes were collected as previously reported (6, 9). In brief, all participants were imaged prior to and following all acupressure treatments. ^1^H-MRS outcomes were acquired on a Philips Achieva 3T system (Best, Netherlands) using an eight-channel receive head coil. For localization of the right posterior insula spectroscopy voxel, we performed T1-weighted 3D-MPRAGE imaging with (0.9 mm)^3^ isotropic voxel resolution. MR spectra were acquired from using 3.0 cm × 2.0 cm × 3.0 cm volumes centered on the right posterior insula using single-voxel point resolved spectroscopy [repetition time (TR)/echo time (TE) = 2,000/35 ms]. Spectra were acquired using “VAPOR” water suppression with 96 averages for each voxel. The spectroscopy data were analyzed using LCModel (Stephen Provencher, Oakville, ON, Canada) (22). The metabolite level analyses utilized two approaches: (1) estimation of metabolite levels expressed as ratios to tCr (combination of Cr and phosphocreatine) and (2) estimation of metabolite levels expressed as concentrations in arbitrary institutional units (AIU) adjusted for cerebrospinal fluid (CSF) content. We focused on two metabolites, Glx and Cr/tCr, the only brain molecules within the posterior insula that we had previously found to be associated with persistent cancer-related fatigue (6). For the estimate of Glx levels, we calculated absolute concentrations, expressed in AIU, using the water signal for normalization (22). Since our voxels incorporated CSF and the volume of CSF dilutes ^1^H-MRS-derived metabolite values, we corrected our metabolite levels for CSF volume for each participant. To adjust for CSF volume Voxel Based Morphometry, a toolbox which operates within the image analysis program Statistical Parametric Mapping (SPM^1^) was used. Corrected metabolite concentrations were entered into IBM SPSS, Windows version v 20 (Chicago, IL, USA) for calculation of differential acupressure treatment effects (post − pretreatment) and correlational analyses with clinical outcomes of fatigue and sleep disturbance.

##### Analysis

We report our ^1^H-MRS mean values and SD comparing treatment changes (post − pre) for posterior insula Glx and Cr/tCr using independent sample *t*-tests on metabolite change scores to analyze between treatment differences (relaxing versus stimulating acupressure). Changes were reported to be significantly different between groups for *p* < 0.05 (two-tailed). All ^1^H-MRS data followed a normal distribution. ^1^H-MRS data were entered into and analyzed with IBM SPSS, Windows version v 21 (SPSS, Chicago, IL, USA).

#### Functional Connectivity Magnetic Resonance Imaging

Participants were scanned on the same 3T Philips Achieva scanner (Best, Netherlands) using an eight-channel head coil identical as for ^1^H-MRS. Ten minutes of resting state fMRI data was acquired using a custom T2* weighted spiral-in sequence [TR = 2,000 ms, TE = 30 ms, flip angle (FA) = 90°, matrix size 80 × 80 with 30 slices, field of view (FOV) = 217 cm, 2.75 mm × 2.75 mm × 4 mm voxels and 300 volumes] followed by a second T1-weighted high-resolution 3D-MPRAGE structural scan for normalization (TR = 9.78 ms, TE = 4.59 ms, FA = 90°, FOV = 219 mm, matrix size 240 × 240 matrix with 150 slices and 0.83 mm × 0.83 mm × 1 mm voxels). During the resting state scan fMRI participants were instructed not to focus on any particular task and stay awake with their eyes open looking at a fixation cross. Since cardiac and respiratory fluctuations are known to influence brain connectivity within several networks (23), participant physiological data were collected simultaneously using a chest plethysmograph for respiratory and infrared pulse oximeter on participants finger for cardiac data. Only participant functional data of less than 2 mm of translation and less than 1° rotation head motion inside the scanner were included for the fcMRI analysis. Whole brain coverage was achieved including the midbrain and rostral brainstem.

##### Preprocessing

Functional connectivity magnetic resonance imaging data were preprocessed using SPM (SPM8; Wellcome Department of Cognitive Neurology, London, United Kingdom) running on MATLAB 7.10 (Mathworks, Sherborn, MA, USA). Upon collection of resting state fcMRI data, physiological artifacts were removed using a custom Matlab algorithm and slice time corrected using FSL 4.1.9 (FMRIB’s Software Library^2^) software. Preprocessing steps included motion correction, realignment, coregistration, normalization to the Montreal Neurological Institute template, and smoothing (FWHM Gaussian kernel of 8 mm) using SPM8.

##### Seed Connectivity Analysis

Seed to whole brain functional connectivity analysis was performed using the *Conn* (Cognitive and affective neuroscience laboratory, Massachusetts Institute of Technology, Cambridge, MA, USA) functional connectivity toolbox (24). The seed region chosen was a 3.0 cm × 2.0 cm × 3.0 cm voxel centered on the right posterior insula as this voxel showed increased levels of Glx and Cr/tCr in cancer survivors in our previous ^1^H-MRS study (6). White matter, CSF, and motion parameters were entered into the analysis as covariates of no interest. A band pass filter (frequency window: 0.01–0.1 Hz) was applied to remove linear drifts and high frequency noise from the data. First-level analysis included bivariate correlations between voxels within the seed region and all voxels throughout the whole brain, thereby creating connectivity maps for each individual. These connectivity maps were then used in group-level analyses comparing changes (post − pre) in acupressure treatment differences (relaxing versus stimulating acupressure) in connectivity in SPM8 using a Flexible Factorial Model. The resulting maps were thresholded at a whole brain *p* < 0.001 uncorrected voxel threshold and *p* < 0.05 family-wise error (FWE) cluster corrected for multiple comparisons. Correlation of brain connectivity outcomes to participant clinical symptoms (fatigue and sleep disturbance) was achieved by obtaining the average fisher transformed *r* values of the resulting significant clusters using the Marsbar toolbox (25), and then performing non-parametric correlations (using Spearman’s Rho) with changes in behavioral measures (post − pre; BFI and PSQI) in SPSS 20 (Statistical Package for the Social Sciences, IBM Corp., Armonk, NY, USA). All connectivity values followed normal distributions. Statistical significance was set at a *p*-value of 0.05 two-sided.

##### Independent Component Analysis (ICA)

Group ICA was performed using the Group ICA of Fmri Toolbox (GIFT) toolbar (26). Component estimates were validated using ICASSO software (27) for 10 iterations to ensure the reliability of ICA algorithm and to increase the robustness of the results. The only component analyzed was the DMN as we found connectivity to this network to be altered in cancer survivors with persistent fatigue, using an identical analytical approach (9). Participant-specific spatial maps and time courses were back reconstructed using spatiotemporal regression (STR) or dual regression options available in GIFT. STR regresses (i) the original participant data onto the combined ICA spatial maps to estimate participant specific time courses for each component; (ii) then regresses the individual participant data back onto these time course matrices to estimate participant-specific spatial maps. Thus, the original combined spatial map and the later estimated spatial maps represent the best approximation for the individual participant specific *Z*-score component maps. These *Z* values reflect the degree of connectivity between each voxel and the group averaged time course of the DMN. The DMN resting state network was identified by spatial correlation with the DMN template provided previously (28, 29). The individual DMN resting state network maps were then passed onto group second-level analyses in SPM8 where differences in resting state network connectivity (post − pre) between treatments (relaxing versus stimulating acupressure) were assessed using a Flexible Factorial Model. For all ICA analyses, significant clusters were identified by thresholding resultant brain maps at *p* < 0.001 uncorrected voxel threshold and *p* < 0.05 FWE cluster corrected significance for multiple comparisons. *Z*-scores from brain regions connected to the DMN showing differential treatment changes in connectivity were extracted, as they were for seed-based connectivity results, tested for normality (all were within a normal distribution), and correlated with clinical outcomes (fatigue and sleep disturbance) and other significant connectivity findings using SPSS v20.

#### Statistical Analysis Plan for Clinical Characteristics

Baseline sociodemographic and clinical characteristics are reported using mean values and SD for continuous variables, and counts and percentages for categorical variables. Comparison between participants in the two acupressure groups on baseline characteristics was tested using independent sample *t*-tests for continuous variables and Pearson chi-square tests for categorical variables. A significance level of *p* < 0.05 (two-sided) was used.

## Results

### Demographics and Acupressure Effects on Clinical Symptoms

19 participants from a clinical trial of acupressure for fatigue in BC survivors (15) participated in both ^1^H-MRS and fcMRI prior to and following the 6 weeks of treatment (relaxing acupressure *n* = 9; stimulating acupressure *n* = 10). There were no differences in age, time since diagnosis, race, ethnicity, cancer stage, initial hormone therapy use, chemotherapy, or radiation between the two acupressure groups (Table 1). Improvements were seen in both fatigue and sleep following treatment for the entire cohort [fatigue mean (SD) change post − pre: −1.81(1.54), *p* = 0.001; sleep mean (SD) change post − pre: −2.17(3.37), *p* = 0.014], but there were no significant differences in symptom improvements between acupressure groups (fatigue *p* = 0.27, sleep *p* = 0.86).

**Table 1:** Demographics of participants by acupressure group.

Demographics and cancer treatments	Relaxing (*n* = 9)	Stimulating (*n* = 10)	*p*-Value
Age mean (SD) years*	58.89 (5.80)	60.10 (8.57)	0.73
Race (*n*)**			0.26
Caucasian	9	8	
African American	0	2	
Ethnicity (*n*)**			0.21
Non-Hispanic	7	10	
Unknown	2	0	
Time since Dx (years)*	6.49 (2.27)	5.13 (1.19)	0.11
Stage (*n*)**			0.44
LCIS	0	1	
DCIS	2	2	
Stage I	3	2	
Stage II	2	4	
Stage III	2	0	
Unsure	0	1	
Initial hormone therapy (*n*)**			0.35
Yes	5	8	
No	4	2	
Chemotherapy**			0.97
Yes	4	4	
No	4	5	
Radiation**			0.07
Yes	2	7	
No	7	3	

*LCIS, lobular carcinoma in situ; DCIS, ductal carcinoma in situ*.

*p-Value determined by independent sample t-test comparing demographics by group.

**p-Value determined with chi square comparing relaxing and stimulating acupressure.

### ^1^H-MRS Levels of both Glx and Cr/tCr Do Not Change with Acupressure

Because our previous study found the levels of both Glx and Cr/tCr to be elevated in the posterior insula in BC survivors with fatigue, we assessed changes in the levels of Glx and Cr/tCr in the posterior insula both before and following both acupressure treatments. The Glx and Cr/tCr levels within the posterior insula did not change significantly following either relaxing (all *p* ≥ 0.82) or stimulating acupressure treatment (all *p* ≥ 0.19), nor were there any differences between groups for changes in metabolite levels (all *p* ≥ 0.24).

### Neurometabolite Levels Are Associated with Sleep Improvement

Although we did not detect any mean differences in neurometabolite levels following acupressure, post minus pretreatment difference scores in posterior insula Glx levels were significantly associated with improvements in sleep for both relaxing (Glx concentration: rho = 0.81; *p* = 0.02) and stimulating (Glx concentration: rho = 0.74; *p* = 0.01; Glx/Cr ratio: rho = 0.82; *p* = 0.003) acupressure. There was a trend for reduced Glx to be associated with less fatigue following stimulating (Glx concentration: rho = 0.59; *p* = 0.08; Glx/Cr ratio: rho = 0.56; *p* = 0.09) but not relaxing (Glx concentration: rho = 0.07; *p* = 0.87; Glx/Cr ratio: rho = 0.18; *p* = 0.64) acupressure. There were no significant correlations between treatment changes in Cr/tCr and either fatigue or sleep (all *p* > 0.05).

### Differential Effects of Relaxing and Stimulating Acupressure on Posterior Insula Seed-Based Functional Connectivity

**Figure 1:**
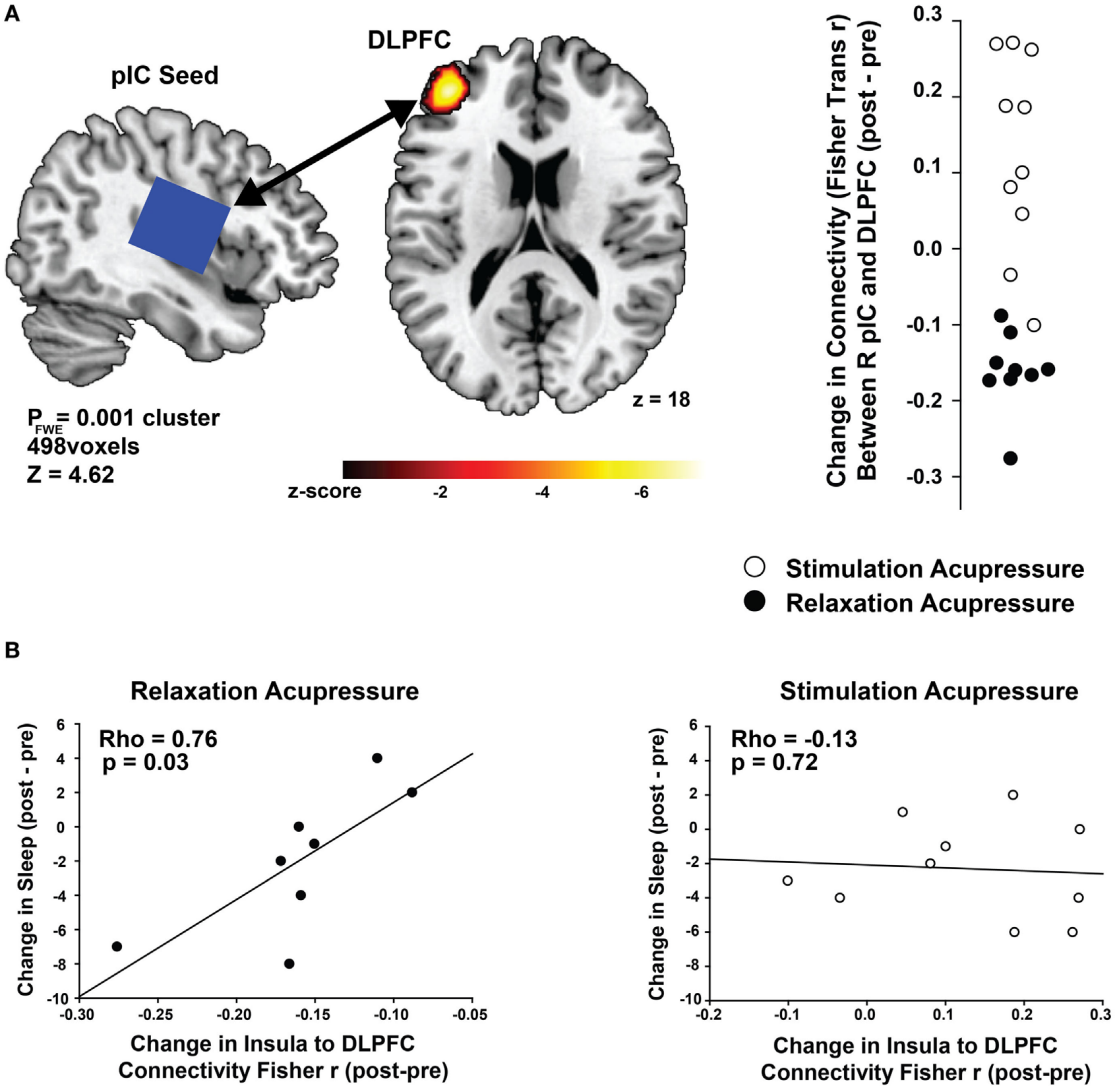
Differential changes in posterior insula (pIC) to dorsolateral prefrontal cortex (DLPFC) connectivity following acupressure. **(A)** Seed-based resting state functional connectivity magnetic resonance imaging analyses show treatment differences (stimulation: open circles, relaxation: black circles) between the posterior insula (seed region; used in proton magnetic resonance spectroscopy) and the DLPFC. Relaxation acupressure reduces insula–DLPFC connectivity whereas stimulation acupressure increases connectivity between these two structures. **(B)** Decreases in posterior insula–DLPFC connectivity following relaxation, but not stimulation, acupressure are associated with improved sleep.

Connectivity between the right posterior insula seed and the left dorsolateral prefrontal cortex (DLPFC) was increased with stimulating acupressure and reduced with relaxing acupressure [Figure 1A; *p* < 0.001 FWE; Table 2; mean difference (SD) in Fisher transformed *r* (post − pre): stimulating 0.13 (0.13); relaxing −0.16 (0.05)]. In unadjusted analyses, reduction in the insula-DLPFC connectivity was associated with improved sleep quality for relaxing (Figure 1B; rho = 0.76, *p* = 0.03) but not stimulating acupressure (rho = −0.13, *p* = 0.72). No changes in fatigue were significantly associated with changes in insula-DLPFC connectivity for either of the two acupressure treatments (all *p* > 0.10).

**Table 2:** Changes in functional connectivity differ for relaxing and stimulating acupressure treatments.

Region	MNI coordinates (*x* *y* *z*)			*Z*-score	Cluster size (voxels)	*p*-Value

Posterior insula seed-based functional connectivity						
Stimulating > relaxing						
L DLPFC	−37	45	21	4.62	498	*0.001* FWE
Relaxing > stimulating						
N.S.						

**Default mode network functional connectivity with independent component analysis**						

Stimulating > relaxing						
L pulvinar thalamus	−19	−31	15	4.32	161	*0.035* FWE
R pulvinar thalamus	13	−33	13	4.07	148	*0.048* FWE
Relaxing > stimulating						
SC/PAG	−3	−31	−3	3.95	22	*0.003* FWE*

*Significant connectivity relationships are in italics*.

*DLPFC, dorsolateral prefrontal cortex; SC/PAG, superior colliculus/periaqueductal gray; L, left; R, right; FWE, family-wise error; N.S., not significant; MNI, Montreal Neurological Institute*.

*Significant at p < 0.05 with small volume correction.

### Differential Effects of Relaxing and Stimulating Acupressure on Functional Connectivity to the DMN

**Figure 2:**
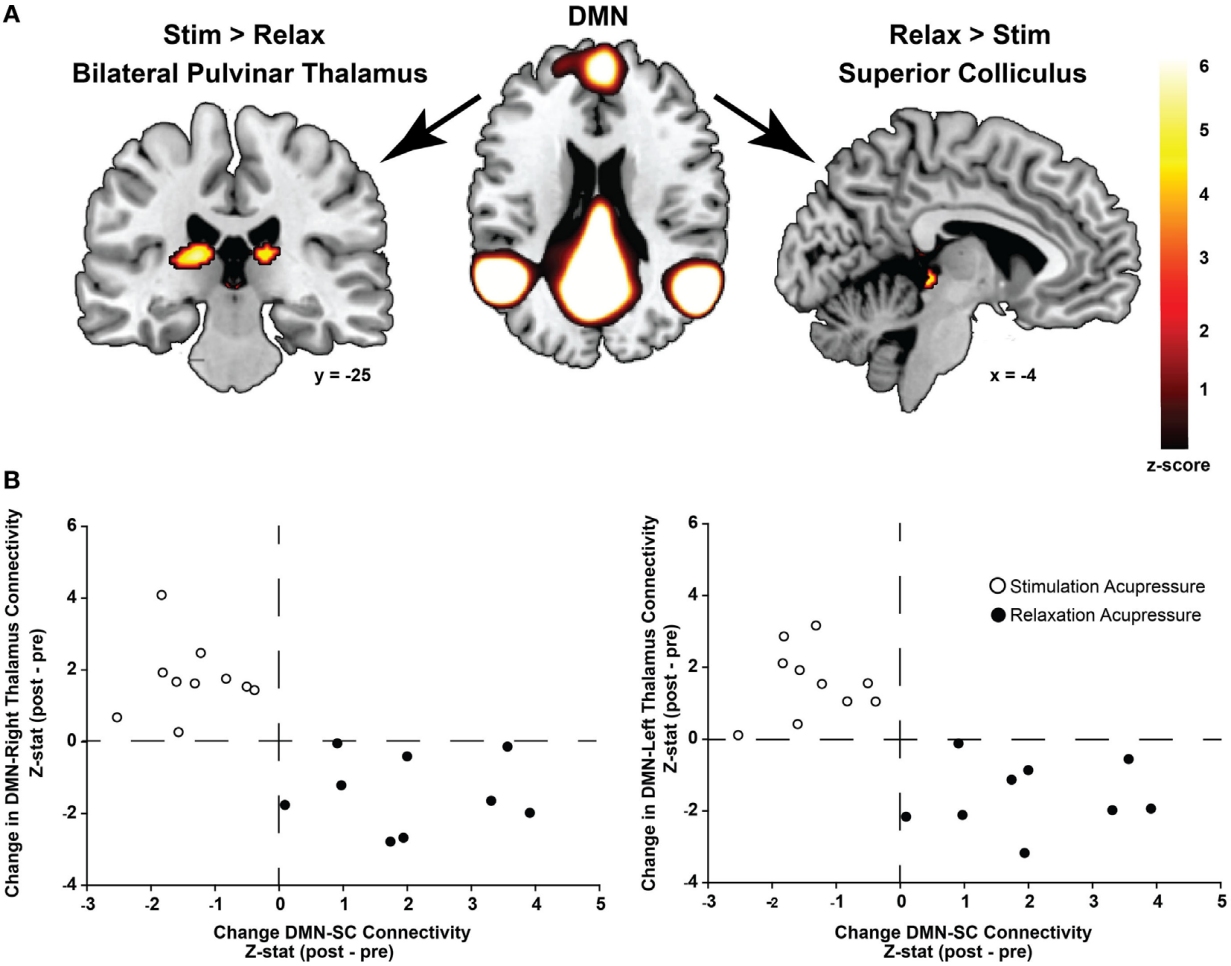
Differential changes in connectivity to the default mode network (DMN) following acupressure. **(A)** Independent component analysis resting state functional connectivity shows increased DMN to bilateral thalamus (pulvinar) connectivity following stimulation acupressure, whereas increased connectivity between the DMN and the superior colliculus (SC)/periaqueductal gray is observed following relaxation acupressure. **(B)** Scatter plots showing changes in pulvinar-DMN connectivity versus changes in SC-DMN connectivity by acupressure treatment (stimulation: open circles, relaxation: black circles). Connectivity patterns are mutually exclusive whereby increases in SC-DMN connectivity are accompanied by decreases in pulvinar-DMN connectivity for relaxation acupressure while the converse is true for stimulation acupressure. This relationship is found in both the right and left thalamus (pulvinar) regions.

Functional connectivity using ICA was performed to identify changes in resting state network connectivity to the DMN contrasting the two different acupressure treatments. Mutually exclusive changes in functional connectivity were found for the two acupressure formulas: relaxing acupressure evoked greater DMN connectivity to the SC–PAG following treatment as compared to stimulating acupressure [Figures 2A,B; Table 2; mean *Z*-score change post − pre (SD): relaxing 2.05 (1.31); stimulating −1.36 (0.66)]. In contrast, stimulating acupressure increased DMN connectivity to the bilateral thalamus (pulvinar) as compared to relaxing acupressure, which decreased this connectivity [mean *Z*-score change post − pre (SD): right pulvinar relaxing −1.4 (1.03); stimulating 1.74 (1.03); left pulvinar relaxing −1.56 (0.96); stimulating 1.57 (0.98)]. These patterns were mutually exclusive, as all participants who received relaxing acupressure had increased DMN-SC/PAG connectivity and decreased DMN-pulvinar connectivity, whereas all participants receiving stimulating acupressure had decreased DMN-SC/PAG connectivity and increased DMN-pulvinar connectivity (Figure 2B). None of these connectivity relationships were associated with changes in fatigue or sleep in unadjusted models (all *p* > 0.1).

## Discussion

We report a novel investigation of dynamic changes in brain functional connectivity patterns that are differentially modulated by two specific self-administered acupressure treatments that are considered to have opposing modes of action from a Traditional East Asian medical perspective (12). Interestingly, we found that breast cancer survivors with persistent fatigue showed decreases in clinical fatigue following acupressure, but there were no significant differences between the acupressure formulas we used. That said, striking differences were observed in their actions on functional activities within the brain, and these effects were associated with persistent cancer-related fatigue markers. This type of a finding is not unheard of in the acupuncture literature as a study examining opioid receptors using positron emission tomography also observed treatment differences in acupuncture effects on the brain, with similar clinical results (30).

We found that connectivity between the posterior insula and DLPFC following relaxing acupressure decreases, while it increases following stimulating acupressure. For relaxing (but not stimulating) acupressure, these changes were associated with improvements in sleep quality. While Glx levels were not independently modulated by either acupressure treatment, reductions in their concentrations were associated with improved sleep for both treatments.

With respect to connectivity to the DMN, we observed a mutually exclusive change in connectivity between this network and the pulvinar (thalamus) and the SC/PAG (brainstem). Relaxing acupressure increased DMN-SC connectivity and decreased DMN-pulvinar connectivity, whereas stimulating acupressure did the exact opposite. While these DMN relationships were not associated significantly with improvements in fatigue or sleep, they depict brain connectivity patterns that are consistent with relaxation acupressure improving sleep and stimulation acupressure enhancing alertness (see below), and importantly they are also consistent with differential subjective symptom findings in our three different trials comparing these same two acupressure formulas (15, 31, 32).

Although there is a large body of literature examining the response of the human brain to acupuncture needle insertion [for meta-analysis see Ref. (33)], the actual demonstration of acupoint “specificity,” namely the differential action of two specific acupuncture point formulas on human patient brain physiology and its relationship to symptom improvement has not been demonstrated [for review of acupoint specificity in general see Ref. (34)]. Previous acupuncture neuroimaging trials that have investigated specificity have either (1) investigated healthy normal participants (35–52), whose brain response is likely different from patient populations, (2) examined the effects of single acupuncture points (35, 36, 40, 42, 43, 47–49, 51), which may not be clinically relevant as acupuncture treatments often involve multiple needle insertions (12), (3) failed to contrast two separate needle formulas and instead compared acupoint stimulation to sham (placebo) acupuncture which does not address specificity (36, 38–40, 42, 44, 46–49, 52, 53), or (4) have not related specific action of treatments to patient symptoms. Our findings here provide direct evidence of differential effects of two acupoint formulas, suggestive of unique treatment mechanisms of action in a clinical population.

Given these divergent processes between acupressure formulas, how might changing connectivity between the posterior insula and the DLPFC be involved in fatigue pathways? There is a large body of literature implicating the insula, and more specifically posterior regions of this structure, in higher order sensory processing (8), alertness (54), and interoception (55). As fatigue is a subjective sensation related to the overall state of energy within the body, an interoceptive alerting action of the insula could be involved in this self-referential appraisal of fatigue. Chronic fatigue research in other populations has also shown that the DLPFC plays a critical role in the “top down” regulation of physical (56) and potentially mental (57) fatigue. Interestingly in these models, the DLPFC receives input from the insula (as well as other brain structures), and this interaction is proposed to either facilitate or inhibit DLPFC action on fatigue (58). We propose that the differential effects of insular connectivity to the DLPFC that our two treatments display may reflect different actions (either reduction of fatigue promoting activity or disinhibition of fatigue reducing activity) on this structure. Although the precise action of these acupoint formulas in fatigue remains to be elucidated, their differential correlation with insula-DLPFC connectivity is at least consistent with the acupressure treatments having somewhat different or even unique actions. Both treatments also showed significant relationships between reduced insular Glx and improved sleep, possibly indicating some overlapping actions; however, this assessment should be made with caution as we did not observe any main effect of either treatment on ^1^H-MRS metabolites.

How might altered DMN connectivity to the pulvinar and to the SC be involved in fatigue pathways and their differential treatment with acupressure? Over the past decade, much attention has been paid to the activity of the DMN. While there is still debate over its exact functions, there is a general consensus that this network is activated when one is not engaged in the external environment and instead is engaged in self-referential thinking (10, 11). A fatigued individual may not be as engaged in the external environment as this is associated with movement/activity (and exacerbation of fatigue) and may be more likely to be thinking about their unpleasant internal condition *via* interoception as mentioned above and thus engaging the DMN. We have also previously reported that connectivity between DMN and the SC/PAG has also been shown to be elevated in non-fatigued breast cancer survivors as compared to those with fatigue (9). This suggests that this connectivity pattern could possibly be “protective” by inhibiting fatigue. Since ablation of the SC in mammals has been shown to profoundly inhibit REM as well as non-REM sleep (59), we speculate that our relaxing acupressure treatment may actually improve sleep, through strengthening DMN-SC connectivity thereby indirectly reducing fatigue by improving sleep.

What does pulvinar connectivity have to do with these distinct acupressure treatments? It has been known for some time that the SC and the pulvinar share both structural and functional connections (60), so it seems reasonable to propose that their functions are linked. Interestingly, the mammalian brain undergoes synchronous alpha wave activity, reminiscent of sleep, when the pulvinar is inhibited (61). In addition, inhibiting pulvinar activity also results in a loss of attention (61). It has been proposed that these actions enable corticothalamic interactions and would be consistent with this structure being required for alertness. Therefore, we speculate that increasing connectivity between the DMN and the pulvinar *via* stimulation acupressure may actually increase alertness and thereby improve daytime fatigue and maybe other symptoms. Our proposed models of action of our two different treatment formulas are consistent with their differential effects on brain functional connectivity namely: relaxation acupressure targets structures that could improve sleep and reduce alertness, and stimulation acupressure acts on regions thought to increase alertness and reduce sleepiness. Importantly, both of these actions could mitigate symptoms of fatigue, just *via different mechanisms*. Of note, this model is also consistent with a large body of work showing that both the pulvinar and the SC are intimately involved in vision and sensitivity to light (62), which likely is also related to sleep and alertness.

Our trial has certain advantages over other brain imaging studies. In addition to being the first study to our knowledge of an MRI study of acupressure, we explored the action of our interventions within a clinical population whose symptom was a target of our treatments. A previous neuroimaging study of acupressure used healthy controls and had imaging outcomes that may not be clinically relevant (63). Also, our study gave participants the ability to treat themselves over the course of multiple weeks, which is in alignment with how acupuncture/acupressure are actually practiced. All participants and study staff were blinded to treatment allocation (except for REH who did not have any patient contact or role in allocation) in essence making this trial double-blinded. Most acupuncture studies require an acupuncturist to perform the treatments and this acupuncturist is difficult to blind since they know when they are giving sham treatments. The staff who trained our participants to perform acupressure had no knowledge about the two treatments making both trainers and participants blinded. Finally, our participants were embedded within a larger randomized controlled trial whose results demonstrate that both these acupoint formulas significantly improve fatigue (as compared to usual care) with the relaxation arm also having significantly more effects on sleep and quality of life (15).

There are certain limitations to our investigation. Our study involved a small number of individuals, so our results most certainly await validation in a larger cohort. Also, our findings may not be generalizable to other fatigued populations as we limited our sample to only breast cancer survivors. Also, all of our participants were female so it is uncertain if these results are also generalizable to males. Finally, most of the women in our trial were white, which limits the generalizability of findings across ethnicity and race.

In conclusion, we provide the first objective evidence that two forms of self-administered acupressure, regarded to have opposing modes of action, act on divergent, often inverse, processes within the brain (connectivity between the insula and DLPFC as well as between the DMN and brainstem structures). Overall, these effects on specific brain regions can be explained through the proposed functions of these areas and are in alignment with the proposed actions of the two treatments. While not conclusive, our findings are consistent with acupoint specificity in acupressure and by extrapolation potentially acupuncture. Looking forward, since there appear to be differing subgroups of fatigued cancer survivors, these data could be used as a mechanistic rationale for personalized acupressure treatments that are specific for individual breast cancer patients.

## Ethics Statement

This study was carried out in accordance with the recommendations of Code of Ethics of the World Medical Association with written informed consent from all subjects in accordance with the Declaration of Helsinki. The protocol was approved by the University of Michigan Medical School, Michigan State University, and Michigan Department of Public Health Institutional Review Boards.

## Author Contributions

RH and SZ contributed to the design of the work. RH, SZ, EI, and TC contributed to data acquisition. RH, EI, CC, JH, TC, NB, and SZ contributed to the analysis and interpretation of data. All the authors contributed to drafting the work and revising it critically and gave final approval of this version. All the authors agree to be accountable for all aspects of the work.

## Conflict of Interest Statement

The authors declare that the research was conducted in the absence of any commercial or financial relationships that could be construed as a potential conflict of interest.

## Footnotes

^1^http://www.fil.ion.ucl.ac.uk/spm/software.

^2^www.fmrib.ox.ac.uk/fsl.
